# To Probiotic or Not to Probiotic: A Metagenomic Comparison of the Discharge Gut Microbiome of Infants Supplemented With Probiotics in NICU and Those Who Are Not

**DOI:** 10.3389/fped.2022.838559

**Published:** 2022-03-07

**Authors:** Jacob A. F. Westaway, Roger Huerlimann, Yoga Kandasamy, Catherine M. Miller, Robert Norton, David Watson, Sandra Infante-Vilamil, Donna Rudd

**Affiliations:** ^1^College of Public Health, Medical and Veterinary Science, James Cook University, Cairns, QLD, Australia; ^2^Centre for Tropical Bioinformatics and Molecular Biology, James Cook University, Townsville, QLD, Australia; ^3^Marine Climate Change Unit, Okinawa Institute of Science and Technology (OIST), Onna, Japan; ^4^Center for Sustainable Tropical Fisheries and Aquaculture, James Cook University, Townsville, QLD, Australia; ^5^College of Public Health, Medical and Veterinary Science, James Cook University, Townsville, QLD, Australia; ^6^Neonatology, Townsville University Hospital, Townsville, QLD, Australia; ^7^Microbiology, Pathology Queensland, Herston, QLD, Australia; ^8^Faculty of Medicine, University of Queensland, Brisbane, QLD, Australia; ^9^Maternal-Fetal Medicine, Townsville University Hospital, Townsville, QLD, Australia

**Keywords:** neonatal, microbiome, preterm (birth), probiotics, gut microbiome, metagenomics

## Abstract

**Background:**

Preterm birth is associated with the development of both acute and chronic disease, and the disruption of normal gut microbiome development. Recent studies have sought to both characterize and understand the links between disease and the microbiome. Probiotic treatment may correct for these microbial imbalances and, in turn, mitigate disease. However, the criteria for probiotic supplementation in NICU's in North Queensland, Australia limits its usage to the most premature (<32 weeks gestation) and small for gestational age infants (<1,500 g). Here we use a combination of amplicon and shotgun metagenomic sequencing to compare the gut microbiome of infants who fulfill the criteria for probiotic-treatment and those who do not. The aims of this study were to determine if probiotic-supplemented preterm infants have significantly different taxonomic and functional profiles when compared to non-supplemented preterm infants at discharge.

**Methods:**

Preterm infants were recruited in North Queensland, Australia, with fecal samples collected just prior to discharge (36 ± 0.5 weeks gestation), to capture potential changes that could be probiotic induced. All samples underwent 16S rRNA gene amplicon sequencing, with a subset also used for shotgun metagenomics. Mixed effects models were used to assess the effect of probiotics on alpha diversity, beta diversity and taxonomic abundance, whilst accounting for other known covariates.

**Results:**

Mixed effects modeling demonstrated that probiotic treatment had a significant effect on overall community composition (beta diversity), characterized by greater alpha diversity and differing abundances of several taxa, including *Bifidobacterium* and *Lactobacillus*, in supplemented infants.

**Conclusion:**

Late preterm-infants who go without probiotic-supplementation may be missing out on stabilizing-effects provided through increased alpha diversity and the presence of commensal microbes, *via* the use of probiotic-treatment. These findings suggest that late-preterm infants may benefit from probiotic supplementation. More research is needed to both understand the consequences of the differences observed and the long-term effects of this probiotic-treatment.

## Introduction

The development of the gut microbiome is an important regulator of lifelong health (1–5). Being born preterm disrupts the gut microbiome's natural development (6). Probiotics are increasingly used as supplements, particularly as adjunctive therapies to prolonged antibiotic treatment. In neonatal intensive care units (NICU) across Australia, probiotic supplementation is becoming the standard of care for the most premature (<32 weeks gestation) and small for gestational age infants (<1,500 g).This is in response to clinical trial validated evidence, that demonstrates effective probiotic supplementation against Necrotising Enterocolitis (NEC) and Late-onset sepsis (LoS) (7–9), in combination with an increased risk of acquiring the disease in very-preterm infants (10). It is now well-recognized that there may be other wide ranging health benefits stemming from early and appropriate gut colonization with bacterial probiotic-species. Although targeted probiotic treatment of the most premature of infants may be justified, those who do not meet the treatment-criteria may be missing out on potential health benefits (11, 12). Preterm infants not supplemented with probiotics may have a disadvantaged start to life (8, 13, 14).

The gut microbiome plays a critical role in the healthy development of the infant, particularly for immunological and metabolic programming (15–17). The disruption of normal gut microbial colonization caused by preterm birth can be associated with acute life-threatening diseases (18, 19), such as necrotising enterocolitis (NEC) and sepsis (20–22). Additionally, a growing body of evidence now suggests disrupted development of the gut microbiome is associated with chronic lifelong conditions, such as asthma (23), type 1 diabetes (24) and metabolic derangements (25). These diseases are more common in those born prematurely, infants who harbor a gut microbiome characterized by low diversity (26) and commensal microbe abundance (27–29), in combination with the presence of a greater number of pathogens (28, 30). Probiotic treatment may provide a solution for improving gut microbiome diversity and commensal microbe abundance, and, in turn, reduce the significant health burden placed on preterm infants.

Despite some heterogeneity between studies reported in the literature (31, 86), probiotics have demonstrated efficacy in reducing the incidence of diseases, such as NEC (7–9), as well as positively modulating the infant gut microbiome (13, 14), in the most premature of infants. The heterogeneity observed could result from the use of different probiotic species (8), variability in the microbiome detection methods used (32) or the many confounding variables that influence the developing gut microbiome. Nonetheless, several countries, such as Japan and Australia, use probiotic-treatment as part of standard care for the most premature of infants, and those at high risk of NEC. Although treatment protocols may vary between countries and neonatal units, here in North Queensland (NQLD) Australia, standard protocol dictates that all infants born <32 weeks gestation and < 1,500 g are supplemented with Infloran^®^, a probiotic containing *Bifidobacterium bifidum* and *Lactobacillus acidophilus*, as approved by the Therapeutic Goods Administration of Australia (33). However, the specificity of this criterion means that preterm infants who fall outside of this criteria, infants who may also suffer from irregular microbial colonization, go without treatment. What significance this has for these non-supplemented preterm infants remains unclear.

Very little is known about the implications of limiting probiotic-treatment to the most premature for the developing microbiome of older preterm infants. Unfortunately, research exploring probiotic-treatment in older preterm infants is lacking, with a 2017 meta-analysis showing the average age for clinical trials is <33 weeks (8). This is not unjustified when considering the previously mentioned inverse correlation of NEC with both gestational age and birth weight (34, 35). Thus, probiotics are targeted at this younger preterm demographic and, in turn, the research as well. Late-preterm infants could be missing out on the benefits provided through probiotic-treatment.

This study was designed to investigate the effect of probiotic treatment on the developing preterm infant gut microbiome, by comparing the gut microbiome of probiotic-supplemented (born <32 weeks gestation) and non-supplemented (born >32 weeks & <37 weeks gestation) preterm infants. The aim of the study was to determine if these two groups have significantly different taxonomic and functional profiles when leaving care, at 36 weeks corrected gestational age. Additionally, we also collected data on known microbiome-covariates so that they could be controlled for using mixed effects modeling. Gut microbiome health at discharge is an important end goal so as to not leave the child at a lifelong disadvantage.

## Materials and Methods

### Study Population

A combination of 16S rRNA gene amplicon and shotgun metagenomic sequencing was used to characterize the microbiome of preterm infants from North Queensland (NQLD), Australia. Where 16S rRNA gene amplicon was applied to the entire cohort, and shotgun metagenomics to a small subset. NQLD is burdened disproportionately by preterm birth, with the North West experiencing the highest rate (12%) of pre-term births (36), and the Torres and Cape the highest proportion (11.7%) of low birth weight infants (36). NQLD also has a large indigenous population, who are more likely to experience prematurity (13%), representing one in ten premature births in Queensland (36). As the prevalence of premature birth in NQLD is increasing, 5% over the last decade (36), the burden that preterm birth places on the families and healthcare system in this region of Australia is significant.

Recruitment sites were the Townsville University Hospital's (TUH) Neonatal Intensive Care Unit (NICU) and Special Care Nurseries (SCN), as well as the Cairns and Hinterland Hospital and Health Service's (CHHHS) SCN. Samples from the probiotic-supplemented infants were all collected from the TUH NICU, as this is the only level six tertiary referral unit in NQLD, which is a specialized unit for dealing with complex pregnancies. All high risk premature infants (<32 weeks gestation and/or <1,500 g) received the probiotic Infloran^®^ (37), containing *Lactobacillus acidophilus* (1 × 10^9^ CFU) and *Bifidobacterium bifidum* (1 × 10^9^ CFU) on a daily basis. Use of this probiotic is approved by the Therapeutic Goods Administration (TGA) of Australia. Infloran^®^ treatment is commenced on the first day of feeding and ceased once the infant is >34–36 weeks gestation. Inclusion criteria for the cohort included: born <32 weeks' gestation and admitted to the NICU at the TUH for the probiotic-supplemented group, and <37 weeks but >32 and admitted to the SCN at the TUH or CHHHS for the non-supplemented group. The exclusion criteria were no parental consent, gestational age of >32 weeks and contraindication to enteral feeds for the probiotic-supplemented group, and no parental consent and gestational age of >37 weeks for the non-supplemented group. Ethics was obtained from the Townsville Hospital and Health Service Human Research Ethics, (HREC/17/QTHS/7). Recruitment and collection were conducted by neonatal nurses, who work in the nurseries, between October of 2017 and October of 2018.

### Sample Collection, Storage, and DNA Extraction

Collection was carried out just prior to discharge (*x* = 36 ± 0.5 weeks gestation), to capture potential probiotic-induced changes, using collection kits (biohazard bag, sterile swab and storage container), with samples sent *via* pneumatic tube systems to Pathology Queensland for storage at −80°C. DNA extraction for both the 16S rRNA gene amplicon and shotgun metagenomic sequencing was carried out using the Bioline ISOLATE Fecal DNA Kit, which includes mechanical bead-beating (38). Modifications were made in consultation with the manufacturer to increase DNA yield. This included increased beta-mercaptoethanol (from 0.5 to 1% to increase DNA solubility and reduce secondary structure formation), addition of an extra wash step (to improve purity) and decreased elution buffer volume from 100 μl to 50μl (to increase final DNA concentration), for overall increased DNA yield and purity. The extracted DNA was then stored frozen at −80°C.

Clinical information was also collected for downstream analyses. This included both maternal data – antenatal antibiotics, chorioamnionitis (clinically diagnosed), preeclampsia (clinically diagnosed), and diabetes (type 1 & 2) and infant data – mode of delivery (vaginal birth vs. cesarean section), diet, gestation, NEC (stage 2 or greater), sepsis (confirmed through culture), neonatal antibiotics and Retinopathy of Prematurity (ROP) (stage 1 or greater). This information can be seen in Table 1.

**Table 1:** Overview of the demographic data for the preterm-infant cohort that underwent 16 rRNA gene amplicon sequencing.

**Categorical variables**
**Variables**	**Levels**	**Count**	**%**
Probiotic supplementation	Yes	63	67.0
	No	31	33.0
Diet	Formula	23	24.5
	Breastmilk	38	40.4
	Formula and Breastmilk	33	35.1
Delivery	Vaginal	32	34.0
	Cesarean	62	66.0
NEC	Yes	5	5.3
	No	89	94.7
Sepsis	Yes	3	3.2
	No	91	96.8
Antenatal antibiotics	Yes	52	55.3
	No	42	44.7
Neonatal antibiotics	Yes	83	88.3
	No	11	11.7
Chorioamnionitis	Yes	28	29.8
	No	66	70.2
Preeclampsia	Yes	13	13.8
	No	81	86.2
Maternal diabetes	Yes	19	20.2
	No	75	79.8
**Continuous variables**			
**Variable**	**Mean/median**		
Gestational age at birth	30.8/30.1 weeks		
Gestational age at collection	36.0/36.0 weeks		

### 16S RRNA Short Amplicon Sequencing

The Illumina metagenomics library preparation protocol was used for library preparation (39), using the Index Kit v2 C (40), along with Platinum™ SuperFi™ PCR Master Mix (41). Sequencing was performed on the Illumina MiSeq system using the MiSeq Reagent Kit V3 600 cycles (40), targeting the V3 and V4 regions with the S-D-Bact-0431-b-S-17/S-D-Bact-0785-a-A-21primer combination (39). Pre-analytical bioinformatics were conducted in *R Studio* Version 3.6.1 (42) with a pipeline adapted from *Workflow for Microbiome Data Analysis: from raw reads to community analyses* (43), which can found under *Additional File 1* or at https://github.com/JacobAFW/SCN_vs_NICU_probiotic_study. *DADA2* (44) was used for quality filtering and trimming, demultiplexing, denoising and taxonomic assignment (using the SILVA Database), and the *microDecon* package (45) used to remove homogenous contamination from samples using blanks originating in extraction.

### Shotgun Metagenomics

A subset of the samples (*n* = 6) was selected on the basis of suitability for shotgun metagenomics analysis (performed by Microba Life Sciences), with samples with the highest extracted DNA concentrations chosen. Six samples, three from probiotic-supplemented and three from non-supplemented infants, were chosen to make species-level and functional comparisons. Other demographic data specific to these infants can be found in Supplementary File 2. These selected samples were shipped to Microba on dry ice. Sequencing was conducted on the Illumina NovaSeq6000 system with 300 bp, paired-end reads. Microba provided an end-to-end service, also conducting the bioinformatics and statistical analysis. This was done using Microba's Metagenomics Analysis Platform (MPA), which includes the Microba Genome Database, the Microba Community Profiler, and the Microba Gene and Pathway Profiler (46). Microba's MPA produces taxonomic and functional profiles. Functional profiles include Enzyme Commission (EC) Number, Membrane Transport Proteins (TCDB) and MetaCyc (database) Pathways and MetaCyc Groups.

### Statistical Analyses

To assess the difference between the probiotic and non-supplemented preterm infants across the entire cohort, while accounting for known associates to the infant gut microbiome, we assessed alpha diversity, beta diversity and taxonomic abundance using mixed effects models. The covariates included; maternal antibiotics (47), maternal diabetes (48, 49), chorioamnionitis (50), preeclampsia (51), maternal diabetes (48), mode of delivery (50, 52), infant diet (53, 54), gestational age, NEC (55, 56), infant sepsis (57, 58), neonatal antibiotics (59) and ROP (60). For beta diversity, we performed an *EnvFit* analysis from the *Vegan* package (61), which compares the differences in the centroids relative to total variation. A Bray-Curtis dissimilarity matrix (62) based on data normalized through Total Sum Scaling (TSS) (63) was used for the *EnvFit* analysis. The significance was based on 10,000 permutations and was transformed using the Benjamini-Hochberg (BH) procedure (64).

For alpha diversity (Shannon Index), we used the package *lme4* (65) to perform a generalized linear mixed effects model. Diversity was calculated at the ASV level. Multicollinearity was assessed using the *AED* package (66) and collinear variables removed. Backwards selection (67) was implemented to find the least complex, yet adequate, model. Significance was determined using an analysis of deviance (Type II Wald Chi-square test) from the *car* package (68), and subsequent *post-hoc* pairwise Tukey comparisons, correcting for multiple comparisons, using the *emmeans* package (69).

*DESeq2* (70), which uses a negative binomial generalized linear model and variance stabilizing transformation, was used for comparing taxonomic abundances between probiotic and non-supplemented groups. Taxa were agglomerated and assessed at the genus level. To identify taxa that were significantly differentially abundance, a Wald Test with the BH multiple inference correction was used. The pre-analytical bioinformatics and statistical analyses can be found in the GitHub link in the Supplementary Material.

To compare probiotic supplemented and non-supplemented infants in the subset of the cohort that underwent shotgun metagenomics, comparisons were again made for alpha diversity, beta diversity and taxonomic abundance. Standard *t*-tests were used for comparing alpha diversity (richness and the Shannon Index), Redundancy analysis (multiple linear regression) for beta diversity, and ALDEx2, with a Welch's *t*-test, for differential abundance. *P*-values were corrected with the BH procedure.

## Results

The aim of the study was to determine if probiotic-supplemented (born <32 weeks gestation) and non-supplemented (born >32 weeks & <37 weeks gestation) preterm infants have significantly different taxonomic and functional profiles when leaving care, at 36 weeks corrected gestational age. The study recruited 94 preterm infants, 63 of which were supplemented with probiotics and 31 not supplemented (other cohort demographic data are available in Supplementary File 2), and collected 94 stool samples (one for each infant). All samples underwent 16S rRNA gene amplicon sequencing, and a subset, 3 probiotic-supplemented and 3 non-supplemented, also underwent shotgun metagenomics.

### 16S RRNA Gene Amplicon Sequencing Analysis

**Figure 1:**
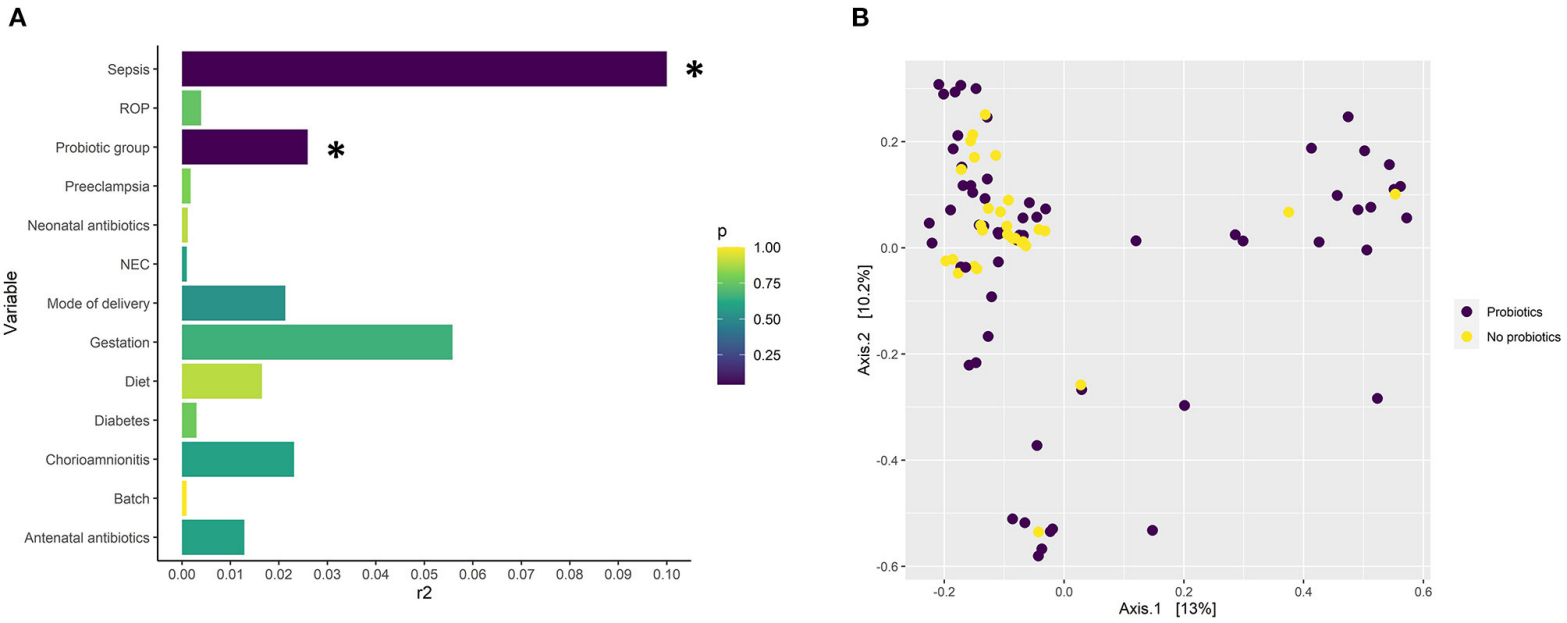
**(A)** Significance and the amount of variance in gut microbiome composition explained by several microbiome covariates modeled with EnvFit on an NMDS ordination based on Bray-Curtis distances from the 16S rRNA short amplicon sequencing data. The x axis describes the explained variance (r^2^) and the color the *p*-value (adjusted for false discovery rate with the Benjamani-Hochberg method). Annotation for necrotising enterocolitis, NEC; retinopathy of prematurity, ROP. *P* < 0.05 = *. **(B)** Principle coordinate analysis (PCoA) plot based on ASV level taxonomy obtained through 16S rRNA short amplicons sequencing describing the dissimilarity of probiotic-supplemented (*n* = 63) and non-supplemented groups (*n* = 31) based on taxonomy.

**Figure 2:**
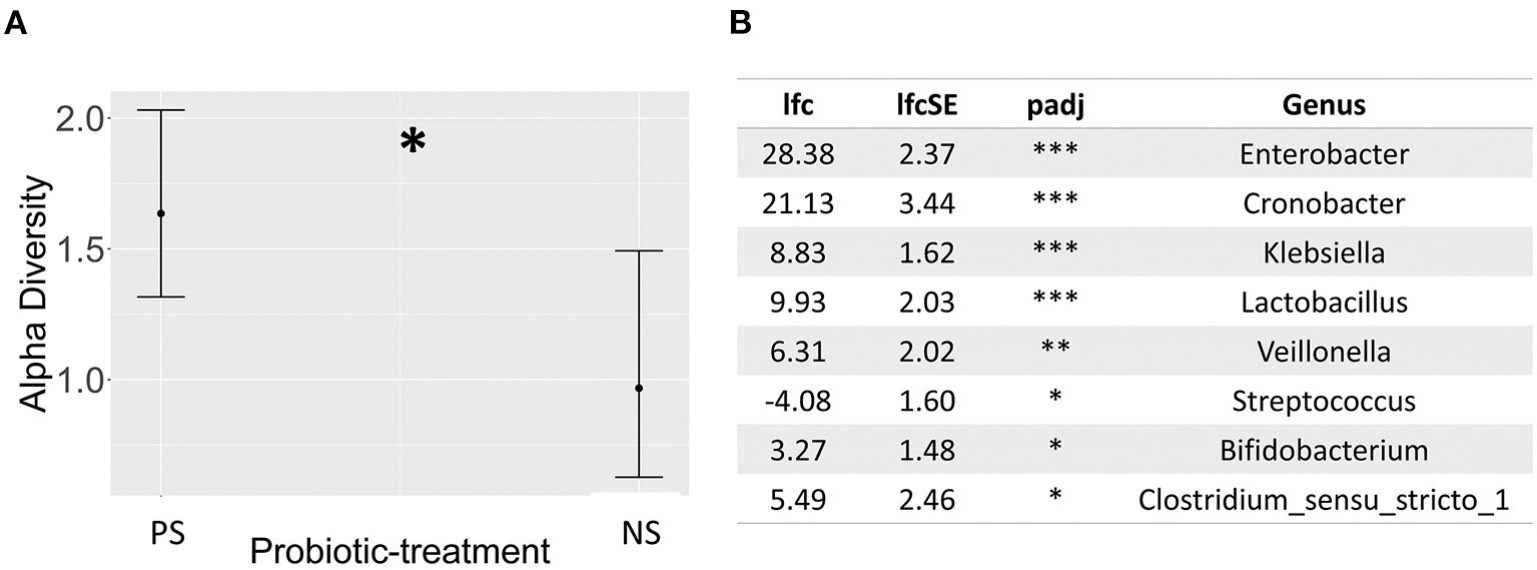
**(A)** Dot whisker plot of the estimates for the probiotic-treatment covariate resulting from a generalized linear mixed effects regression model, exploring the effect of several known microbiome covariates on the Shannon diversity index derived from 16S rRNA short amplicon sequencing, and based on ASVs, **(B)** Table describing significantly differentially abundant taxa between probiotic-treated (base-level) and non-treated infants, using 16S rRNA short amplicon sequencing, as determined by DESeq2 analysis, based on data transformed through DESeq2's variance stabilizing transformation. Annotation for probiotic-supplemented, PS; non-supplemented, NS; p-adj: adjusted *p*-value; lfc, log2-fold change; lfcSE, log2-fold change standard error; NT. *P* < 0.05 = *, *P* < 0.01 = **, *P* < 0.001 = ***. Sample sizes; probiotic supplemented = 63, and non-supplemented = 31.

16S rRNA gene amplicon sequencing showed probiotic treatment influences the preterm-infant gut microbiome, having a significant effect across all three metrics measured: alpha and beta diversity, and taxonomic abundance. Probiotic supplementation, along with sepsis, were the only covariates found to have a significant association (*P*< 0.05) with ASV level bacterial profiles (Figures 1A,B), with sepsis explaining more variation (*r2* = *0.33*). In addition, infants supplemented with probiotics had significantly higher alpha diversity (*P*< 0.05) than non-supplemented infants (Figure 2A), as well as significantly differential abundance of several taxa. This included higher abundance of *Enterobacter, Cronobacter, Klebsiella, Veillonella and Clostridium Sensu Stricto 1*, as well as the probiotic-genera *Bifidobacterium* and *Lactobacillus*, and lower abundances of *Streptococcus* (Figure 2B). *Bifidobacterium* and *Lactobacillus were* observed in 55 and 39 of the 63 discharge infants, in contrast to 10 and 6 in the non-supplemented group (Supplementary File 2).

### Shotgun Metagenomic Sequencing Analysis

The results from the shotgun metagenomics showed that there was a high rate of colonization with *Enterococcus faecalis*, as well as other aerobic species from the Proteobacteria phylum across all samples. Infants were also commonly colonized with skin dwelling microbes, such as *Streptococcus* spp., *Staphylococcus* spp., and *Veilonella* spp. However, despite these cohort-wide trends, probiotic supplementation still appeared to have some effect on the gut microbiome. Although, not significantly different, samples clustered by supplementation-group for species-level taxonomy, MetaCyc pathway, MetaCyc group and EC number profiles (Supplementary File 2), suggesting distinct taxonomic and metabolic profiles. Due to the small sample size, we were unable to account for other covariates with the shotgun metagenomics analysis. However, no infants in this subset were diagnosed with sepsis, the only other significant covariate identified through 16S metabarcoding. Alpha diversity metrics were supportive of what was observed at the genus level (Supplementary File 2), with significantly higher species-level alpha diversity in probiotic supplemented infants (Shannon index, *P*< 0.05), which also translated into significantly higher MetaCyc pathway diversity (Richness, *P*< 0.05).

**Figure 3:**
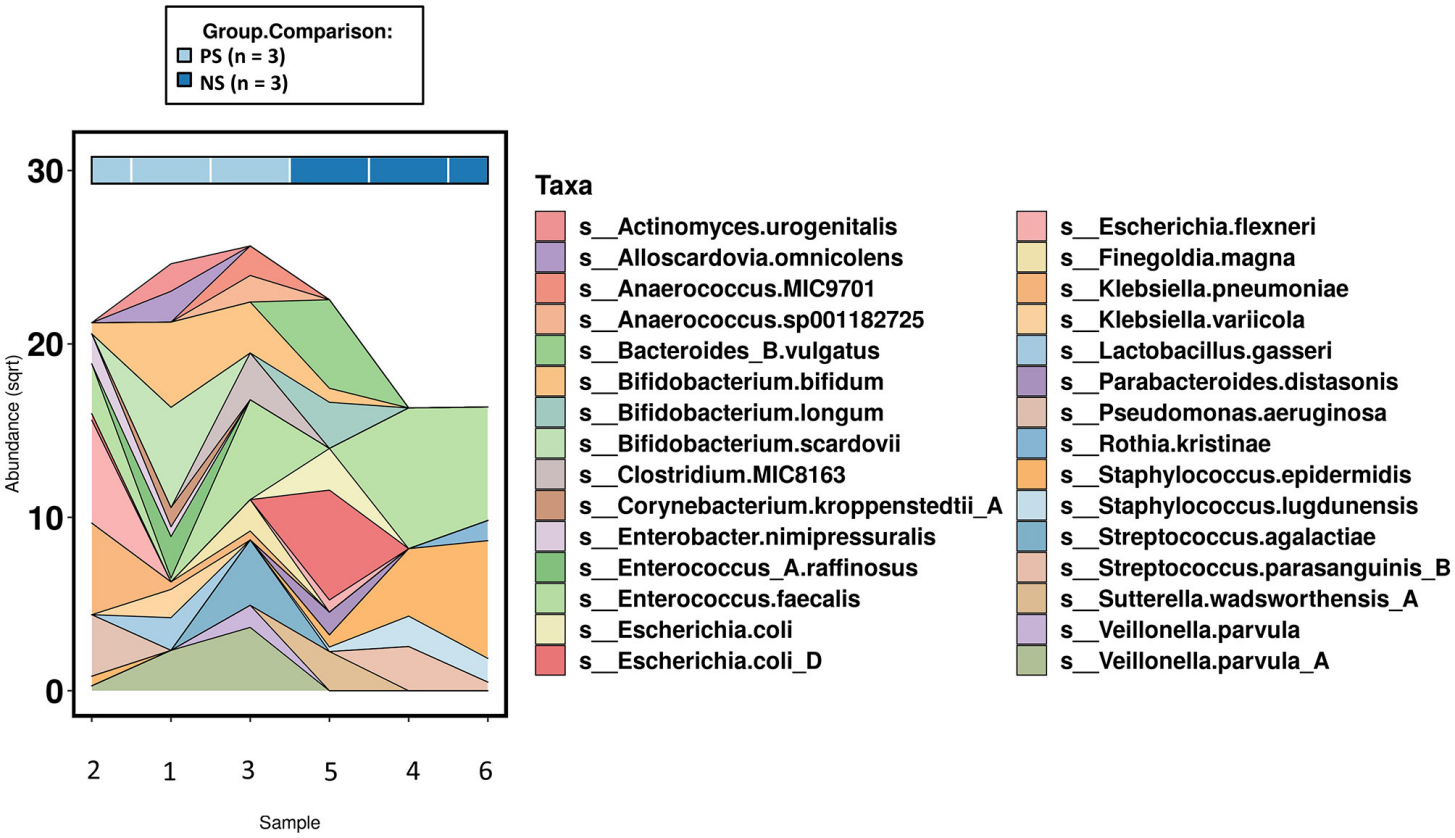
Area chart of species level abundances (top 30-most abundant)across the subset of samples that underwent shotgun metagenomics (*n* =6). PS, probiotic-supplemented; NS, not supplemented; sqrt, square root transformation.

When comparing species and functional profile abundance between the probiotic-supplemented and non-supplemented groups, there were no significant differences when adjusting for multiple comparisons. However, there were several taxa that were only present in one group or the other, resulting in their ranking as top associations. The top associations (by *p*-value) in species level abundances were *Staphylococcus lugdunensis, Veilonella parvula* and *Klebsiella pneumoniae*. *S. lugdunensis* was only observed in non-supplemented infants and the latter two species only in probiotic-supplemented infants (Figure 3), supporting what was observed at the genus level. Additionally, probiotic-supplemented infants showed a different probiotic species colonization pattern compared with non-supplemented infants. With the exception for *B. bifidum* and *B. longum* in a single non-supplemented infant, no *Lactobacillus* spp. or other *Bifidobacterium* spp. were observed in the non-supplemented infants. In contrast, *Bifidobacterium bifidum* was observed in all three of the probiotic-supplemented infants and one of the non-treated individuals. The species made up 9.8%, on average, of the total species relative abundance in the treated group and only 0.12% in the non-supplemented. *Lactobacillus acidophilus* was observed in only two of the probiotic-treated infants, but with only 0.23% of the total species abundance. Despite the different colonization patterns between the two groups, neither univariate comparison resulted in a significant difference.

**Figure 4:**
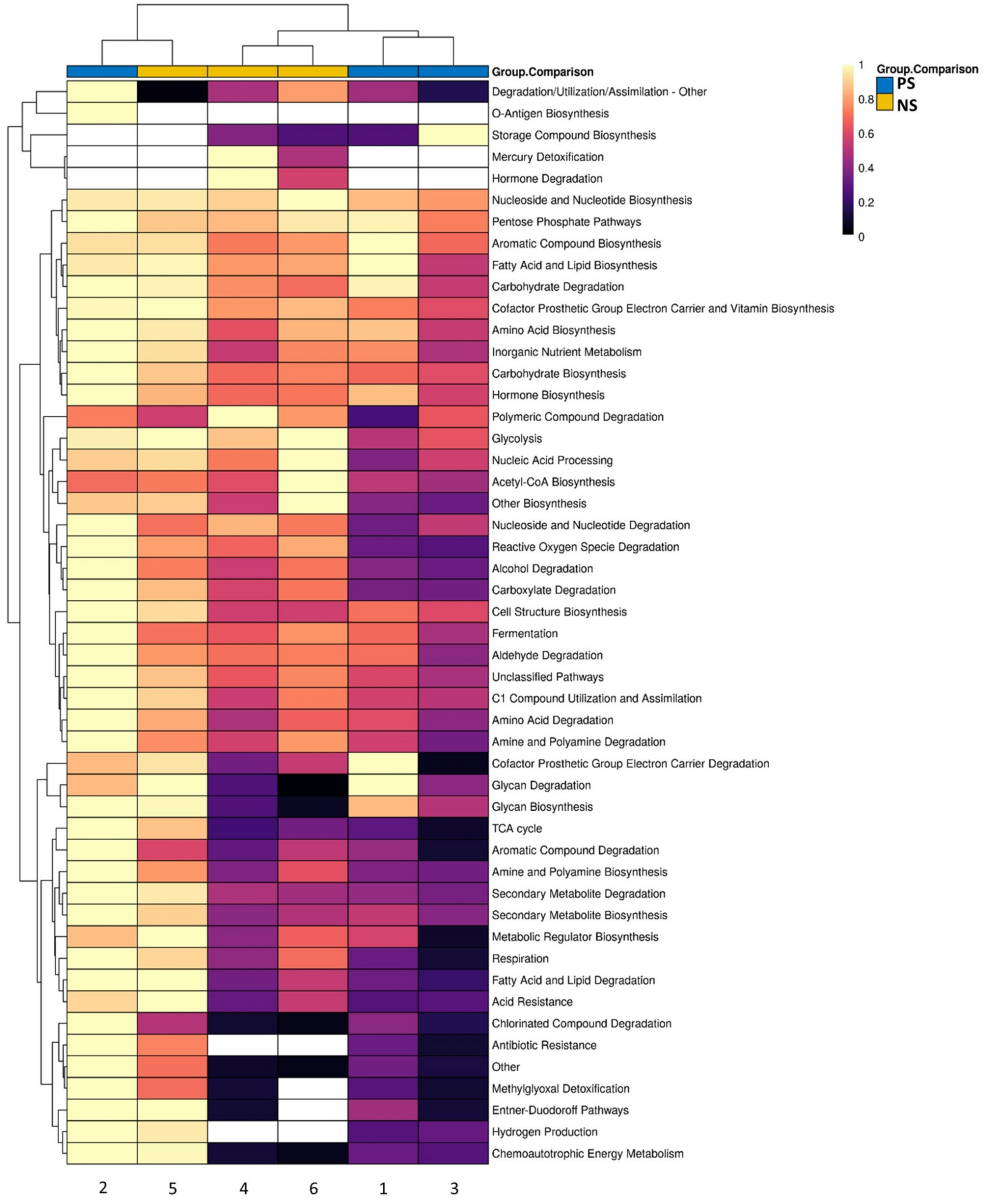
Scaled heatmap of MetaCyc groups for the subset of samples that underwent shotgun metagenomics across probiotic-supplemented (*n* = 3) and non-supplemented (*n* = 3). Color scale shows not-detected (white), and abundances ranging from low (black) to high (yellow).

There were no significant differences between the supplemented and non-supplemented probiotic groups for functional genetic groups. However, there were several note-worthy differences observed within the MetaCyc group profile (Figure 4). This includes the presence of *Antibiotic Resistance* and *Hydrogen Production* groups in all three probiotic-treated infants relative to one non-supplemeneted infant, and the top associations of *Reactive Oxygen Species Degradation* (greater in probiotic-treated) and *Carboxylate Degradation* (greater in non-supplemented) (Figure 4).

## Discussion

We explored the gut microbiome of preterm infants, comparing microbial populations between infants who received probiotic-supplementation and those who did not. Specifically, a combination of 16S rRNA gene amplicon (full cohort) and shotgun metagenomic sequencing (a subset of the cohort) was used to determine if differences exist in the gut microbiome between probiotic-supplementation groups. The results suggest that a significant difference does exist in the bacterial profiles of probiotic-supplemented and non-supplemented preterm infants at discharge from the hospital (36 ± 0.5 weeks gestation), and that these differences in taxonomy may translate into differences in functional profiles. In addition, the probiotic-taxa contained within Infloran^®^ may colonize most infants. Although these findings may currently have limited direct translation in the clinic, they add weight to the argument for expanding the probiotic supplementation criteria in Australia, which is currently limited to those infants born <32 weeks gestation and <1,500 g.

Probiotic treatment groups have distinct microbiomes, characterized by greater alpha diversity in those supplemented.

Probiotic supplementation may contribute to differences in gut microbiome diversity. The results suggest that there was significant variation in the alpha diversity of the gut microbiome between the two groups, with probiotic supplemented infants having significantly greater alpha diversity. This suggests Infloran may be contributing to the establishment of a more diverse, and in turn, healthier gut microbiome. In addition, these results run counter to what one would expect when comparing early- and late-preterm infants not supplemented with probiotics, as lower alpha diversity is typically associated with lower gestational age (26, 30). However, there is evidence to support increased diversity in response to probiotic supplementation in extremely preterm infants (71), which may also be compounded by the widespread use of antibiotics in our cohort, and the ability of probiotics to correct for this (72). With greater gestational age, microbial diversity increases, and the microbiome becomes more stable (73). This increase in diversity is protective against instability (74, 75), meaning protective against overgrowth by opportunistic pathogens, as seen in diseases such as NEC and LoS (21, 76). As a result, non-supplemented late-preterm infants may, therefore, be missing out on protection provided through higher diversity afforded *via* probiotic supplementation. However, it is worth noting that although higher diversity can be indicative of greater microbiome health, it may not always be the case. A prime example of this is the significant association previously shown between breastfeeding and low alpha diversity (53, 77). Thus, caution should be taken when interpreting these results, and more broadly, when using alpha diversity metrics as a proxy for gut microbiome health.

### Higher Rate of Colonization and Abundance of Probiotic-Taxa in Those Supplemented

Taken together, the 16S rRNA short amplicon and shotgun metagenomic sequencing suggest the probiotic-taxa, *Lactobacillus acidophilus* and *Lactobacillus bifidus* (*Bifidobacterium bifidum*), colonize the infant microbiome, but not consistently. This pattern of probiotic-species colonization is supported by previous work (13, 14). The shotgun metagenomic sequencing, that was performed on a subset of the cohort, was able to identify *B. bifidum* across all three supplemented infants and *L. acidophilus* in one. The low level of *L. acidophilus* colonization has been reported previously (13, 14), and unfortunately, may result from poor product-quality. These quality assurance concerns are highlighted by an inability to produce robust quantification of *L. acidophilus* in the past (14), and our 16S rRNA sequencing of the probiotic itself, which found uneven proportions of taxa within the probiotic Infloran^TM^ at the genus level, dominated by *Bifidobacterium* (Supplementary File 2). The cause of these irregularities is unclear, however, this is not the first time irregularities in the microbial profiles of probiotic supplements have been observed (78).

With 16S rRNA short amplicon sequencing, both *Bifidobacterium* (*p*< 0.05) and *Lactobacillus (p*< 0.001) were in significantly greater abundance in the probiotic supplemented group and were observed in 55 and 39 of the supplemented infants, respectively. In contrast we identified only 10 infants with *Bifidobacterium* and 6 with *Lactobacillus* in the non-supplemented group. Although this does not provide direct evidence for widespread probiotic-species colonization, the higher frequency and abundance of these genera suggests that treatment with Infloran^®^ promotes the growth of these commensals, which may aid in the fight against pathogenic infection and in immune and metabolic system development (79, 80). The significance of this greater presence of common commensal microbes in a very-preterm demographic is compounded by the contrasting observations suggesting a negative relationship between birth gestational age and limited or delayed colonization with *Lactobacillus* and *Bifidobacterium* (81, 82). However, not all supplemented infants were colonized by *Lactobacillus* and *Bifidobacterium*. The lack of colonization seen for the *Lactobacillus*, may be due, at least in part, to issues with the probiotic, outlined above. However, the reason for low colonization with *Bifidobacterium*, remains unclear, as no clinical variable included in our analyses had a negative association with either genus, and all probiotic-supplemented infants that had samples collected at > 36 weeks gestation (post-supplementation) still had *Bifidobacterium* present (Supplementary File 2). Whether *Bifidobacterium* colonizes the probiotic-supplemented infant gut may be dependent on the complex interaction of multiple factors.

The greater abundance of *Bifidobacterium* colonization may persist beyond probiotic treatment. As previously mentioned, probiotic treatment for infants at TUH ceases between 34- and 36-weeks gestational age. However, our results suggest that *Bifidobacterium* persists beyond this time point, as the genus was present in all 23 infants with samples collected > 36-weeks gestation. This supports previous studies that have observed long-term probiotic-species colonization, at least with *Bifidobacterium* (14, 83). As moderate to late-preterm infants are at a lower risk of acute disorders than those born very-preterm (34, 35), long-term benefits provided through probiotic-treatment may be more significant for the moderate to late-preterm demographic. Further work needs to be done to explore long-term differences between supplemented early- and non-supplemented moderate/late-preterm infants.

### Probiotic Supplementation Associated With Differences in Non-probiotic Taxa

*Enterobacter, Cronobacter, Klebsiella, Veillonella* and *Clostridium Sensu Stricto 1* were all higher in probiotic supplemented infants, whilst *Streptococcus* had a greater abundance in those not supplemented. The significance of such modulation is unclear, as despite several notable pathogens within these genera, many other species can be considered normal flora. As early-life microbial colonization occurs concomitantly with development of the immune system, immune-system maturation is influenced by the presence of commensal microbes (84), therefore, fewer commensal microbes at this stage of life may be detrimental long-term. Although it is unclear whether these specific taxa play a role in morphological or functional development of the immune system (84), their presence will at least lead to preferential development of immune tolerance, reducing the likelihood of such taxa reaching their “pathogen-potential” later in life. In addition, if these taxonomic differences persist, specifically reduced levels of *Veillonella*, non-supplemented infants may be at a greater risk of chronic diseases like asthma, which has previously been shown to be associated with such differences (23).

At the species level, there were notable differences in *Veilonella parvula, Klebsiella pneumoniae* and *Staphylococcus lugdunensis. S. lugdunensis* was found in all non-supplemented but not in probiotic-supplemented infants, and *V. parvula* and *K. pneumoniae* across all probiotic-supplemented but not in non-supplemented infants. Although this appears to align, in part, to the difference in probiotic-supplemented at the genus level, these differences may be better explained by other variables. For instance, *K. pneumoniae* was one of the most abundant taxa in probiotic-supplemented infants. However, it is possible that this species was selected for through antibiotic treatment (85), which seems likely when considering all three of the probiotic-supplemented infants received antibiotics and that *K. pneumoniae* had the greatest abundance of ABR genes across all species (Supplementary File 3). Unfortunately, the sample size of the shotgun analysis was too small to draw conclusions, and future work should apply shotgun methods to a greater sample size to elucidate why we are seeing differences in given taxa.

### Differences in Functional Profiles Between Treatment Groups

These limited statistically significant taxa, including a negligible, non-significant difference in the probiotic taxa, may not be physiologically insignificant. From both an ecological and physiological perspective, several small changes in what may be critical taxa, may have significant consequences, especially if these differences are in taxa that harbor genes critical to key environmental processes. Although not significant, and as previously mentioned, ABR genes were in higher abundance across the probiotic-supplemented group, whilst only present in a single non-supplemented infant, with Hydrogen Production following the same pattern. The presence of ABR and Hydrogen Production genes in the probiotic-supplemented infants is also closely linked to specific species. The previously mentioned abundances of *K. pneumonia* and *V. parvula*, along with *E. flexneri* (in one infant), were the only species across all probiotic-supplemented infants to have these pathways present. Although this example may not have any significant implications, it highlights the functional importance the presence of a single species can have. Another example of this importance is highlighted by the lower abundance of 1.3-beta-galactosyl-N-acetylhexosamine phosphorylase in the non-supplemented group (Supplementary File 1). The enzyme is a critical component of an enzymatic system within *Bifidobacterium spp*. that metabolizes human milk oligosaccharides. Thus, without species like *B. bifidum*, the non-supplemented infants have less capacity to reap the benefits of breast feeding. Thus, although differences may be subtle and temporary for species like *B. bifidum*, these differences could have larger, long lasting physiological consequences.

### Limitations

This study has several limitations. This includes the different ages in the treatment-groups, the distribution of samples across two sequencing runs, the limited taxonomic depth provided through 16S rRNA gene amplicon sequencing, and the small samples size that underwent shotgun metagenomics. To mitigate the effect of the different ages between the treatment groups, and the batch-effect introduced through multiple sequencing runs, both variables were included in all three of the mixed effects models. As for the limited taxonomic depth and limited sample size of the sub-cohort that underwent shotgun metagenomics, this could be overcome using shotgun metagenomics across the entire cohort. However, this technique, and others of similar resolution, are cost prohibitive at present (32).

## Conclusion

There was a significant difference in overall gut microbiome community composition between probiotic-supplemented and non-supplemented infants, with alpha diversity greater in the supplemented infants. Moderate to late preterm-infants who go without probiotic-supplementation may be missing out on stabilizing-effects provided through probiotic-supplementation, which may help to prevent disease. These results suggest that there could be a role for probiotic supplementation in the treatment of late-preterm infants in North Queensland, Australia. However, caution should be taken when extrapolating from single-center studies to other locations. In addition, rather than provide answers, the differences in taxonomy prompt more questions. Significant differences exist at the genus level, but what are the consequences of these differences? Additionally, differences observed at both the species and functional level highlight the power of shotgun metagenomic sequencing, and we suggest that as the cost of this technology continues to decrease, that future work should adopt this approach. Obtaining species-level and functional profiles in this cohort would provide us with a better understanding of the physiological and ecological consequences of withholding probiotic-treatment from late-preterm infants.

## Data Availability Statement

The datasets presented in this study can be found in online repositories. The names of the repository/repositories and accession number(s) can be found at: https://www.ncbi.nlm.nih.gov/, PRJNA751712. Scripts for the bioinformatics and analyses can be found at https://github.com/JacobAFW/SCN_vs_NICU_probiotic_study.

## Ethics Statement

The studies involving human participants were reviewed and approved by Human Research Ethics Committee from the Townsville Hospital and Health Service. Written informed consent to participate in this study was provided by the participants' legal guardian/next of kin.

## Author Contributions

JW, DR, YK, RH, RN, SI-V, and DW: substantial contributions to conception and design, acquisition of data, or analysis, and interpretation of data. JW, DR, CM, YK, RH, SI-V, and RN: drafting the article or revising it critically for important intellectual content. All authors approved the final version to be published.

## Funding

This work was funded through both a Study, Education and Research Trust Account (SERTA) research grant (Townsville Hospital and Health Service), and a Far North Queensland Hospital Foundation grant. Neither of the funding bodies played a role in the design of the study or collection, nor the interpretation of data and writing.

## Conflict of Interest

The authors declare that the research was conducted in the absence of any commercial or financial relationships that could be construed as a potential conflict of interest.

## Publisher's Note

All claims expressed in this article are solely those of the authors and do not necessarily represent those of their affiliated organizations, or those of the publisher, the editors and the reviewers. Any product that may be evaluated in this article, or claim that may be made by its manufacturer, is not guaranteed or endorsed by the publisher.

## Supplementary Material

Supplementary File 1 Supportive data not included in the manuscript.

Supplementary File 2 MetaCyc Group data within species and across samples.

Supplementary File 3 Enzyme Commission data within species and across samples.

## Abbreviations

ABR: antibiotic resistance
ASV: amplicon sequence variant
B: Bifidobacterium
CHHS: Cairns and Hinterland Hospital and Health Service
DNA: deoxyribonucleic acid
E: Escherichia
EC: Enzyme Commission
EC: Enzyme Commission Number
HMO: human milk oligosaccharide
K: Klebsiella
L: Lactobacillus
LoS: Lat-onset sepsis
MetaCyc: metabolic pathway database
MPA: Metagenomics Analysis Platform
MPA: Metagenomics Analysis Platform
NEC: necrotising enterocolitis
NICU: neonatal intensive care unit
NMDS: Non-Metric Multidimensional Scaling
NQLD: North Queensland
NT: non-treated
PCA: Principal component analysis
PCoA: Principal coordinate analysis
PCR: polymerase chain reaction
PERMANOVA: permutational analysis of variance
PT: probiotic-treated
ROP: retinopathy of prematurity
rRNA: ribosomal ribonucleic acid
SCN: Special care nursery
TCDB: Membrane Transport Proteins
TCDB: Transport Proteins
THHS: Townsville Hospital and Health Service
V: Veillonella.
