# Supplementary material for: To Probiotic or Not to Probiotic: A Metagenomic Comparison of the Discharge Gut Microbiome of Infants Supplemented With Probiotics in NICU and Those Who Are Not
Source: Front Pediatr. 2022 Mar 7;10:838559. doi: 10.3389/fped.2022.838559 (PMC8957066; doi:10.3389/fped.2022.838559)
Supplement: Supplementary File 1 — Supportive data not included in the manuscript. [file Data_Sheet_1.docx]

| **ID** | **1** | **2** | **3** | **4** | **5** | **6** |
| --- | --- | --- | --- | --- | --- | --- |
| **Probiotics** | Yes | Yes | Yes | No | No | No |
| **Diet** | Formula | Formula | Formula | Breastmilk | Breastmilk & Formula | Breastmilk |
| **NEC** | No | Yes | No | No | No | No |
| **Sepsis** | No | No | No | No | No | No |
| **Delivery** | Caesarean | Caesarean | Caesarean | Caesarean | Vaginal | Caesarean |
| **Antenatal antibiotics** | Yes | Yes | No | No | No | No |
| **Neonatal antibiotics** | Yes | Yes | Yes | No | No | No |
| **Chorioamnionitis** | No | Yes | Yes | No | No | No |
| **Maternal diabetes** | No | No | No | No | Yes | No |
| **Preeclampsia** | No | No | No | No | No | No |
| **ROP** | No | Yes | Yes | No | No | No |
| **Gestational age at birth** | 26 | 24 | 25 | 34 | 37 | 34 |
| **Gestational age at collection** | 31 | 36 | 35 | 35 | 39 | 35 |

*Supplementary Table 1. Overview of the demographic data for the six preterm-infants who had samples that underwent shotgun metagenomic sequencing.*


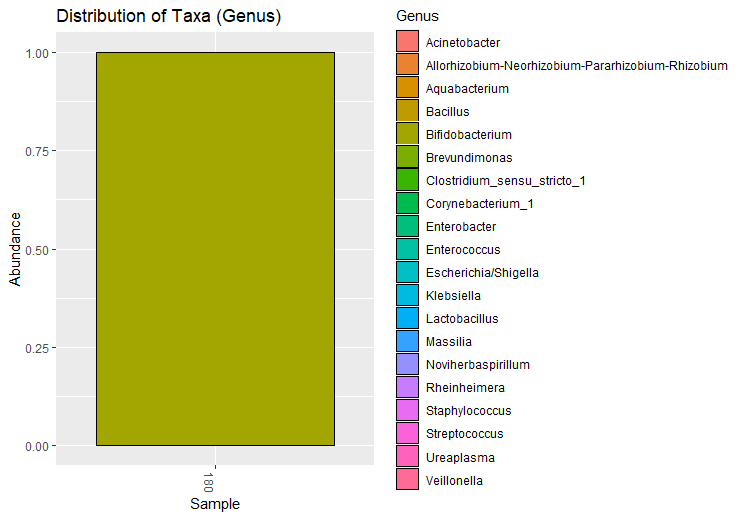


*Supplementary Figure 1. Bar plot of relative abundance of taxa at the genus level from the probiotic Infloran.*

*
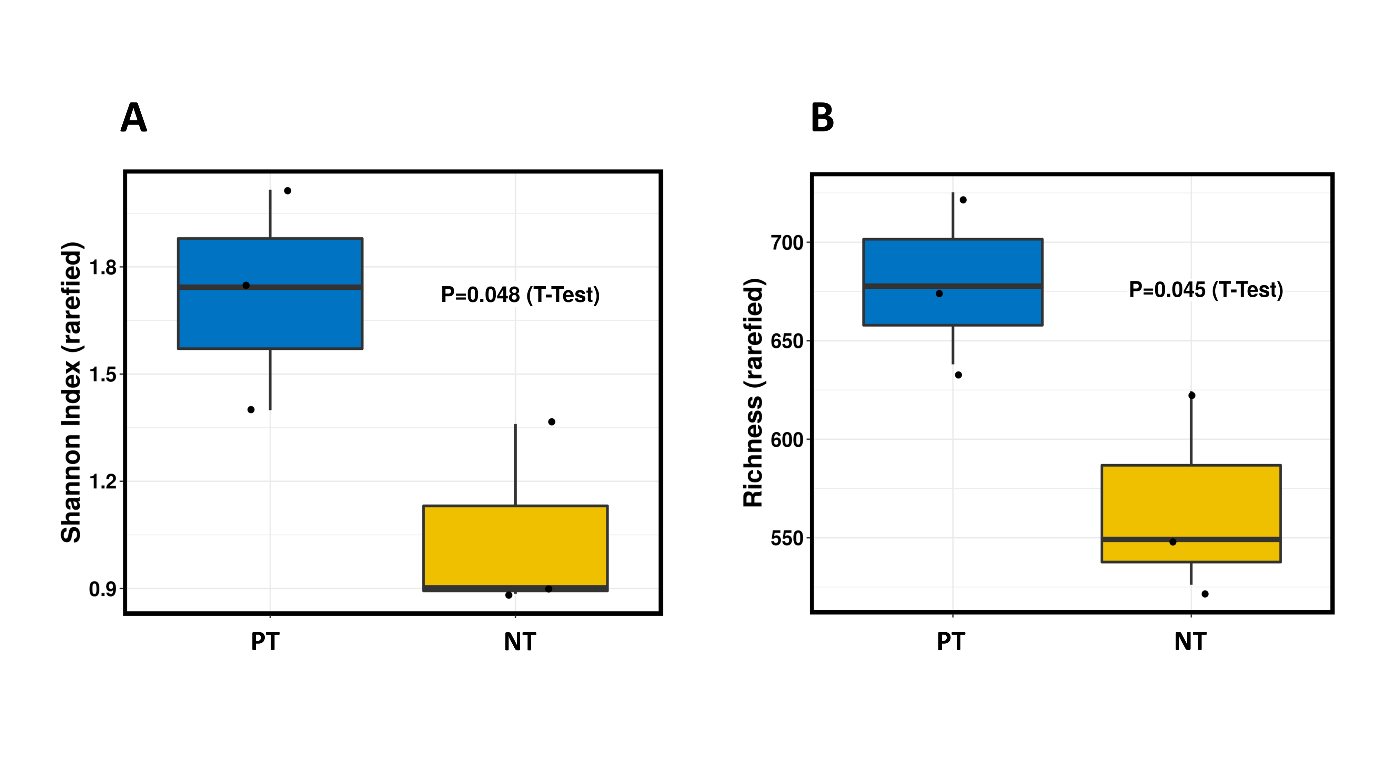
*

*Supplementary Figure 2. A: Boxplots comparing the Shannon Index for probiotic-treated and non-treated infants, B: Boxplots comparing the Richness for probiotic-treated and non-treated infants. Annotation for probiotic-treated; PT: non-treated; NT.*

*
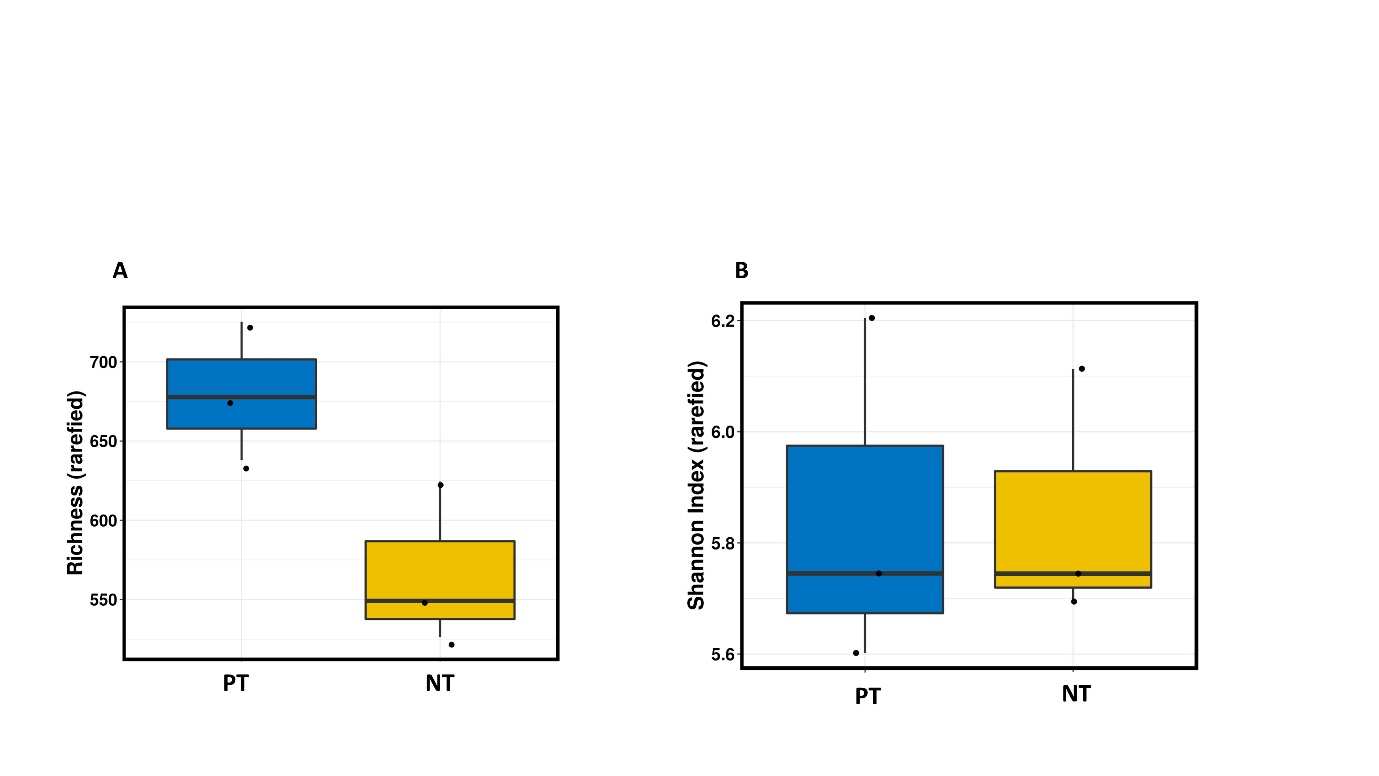
*

*Supplementary Figure 2. A: Boxplots comparing the Richness of MetaCyc Pathways for probiotic-treated and non-treated infants, B: Boxplots comparing the Shannon Index of MetaCyc Pathways for probiotic-treated and non-treated infants. Annotation for probiotic-treated; PT: non-treated; NT.*

| *contrast* | *estimate* | *SE* | *z.ratio* | *p.value* |
| --- | --- | --- | --- | --- |
| Treated – Non-treated | 0.52 | 0.25 | 2.12 | 0.03 |

*Supplementary Table 2. Tukey’s pairwise comparison from generalized linear mixed effects modelling, comparing probiotic-treated to non-treated infants.*

| **envfit.** | | |
| --- | --- | --- |
| *Variable* | *r2* | *p* |
| Gestational_Age_at_Birth | 0.06 | 0.66 |
| **Probitoic_Treatment** | **0.03** | **0.04** |
| Feeding_Type | 0.02 | 0.90 |
| NEC | < 0.01 | 0.59 |
| **Sepsis** | **0.33** | **0.04** |
| Mode_of_Delivery | 0.02 | 0.53 |
| Neonatal_Antibiotics | < 0.01 | 0.90 |
| Chorioamnionitis | 0.02 | 0.59 |
| Preeclampsia | < 0.01 | 0.80 |
| ROP | < 0.01 | 0.74 |
| Batch | < 0.01 | 1.00 |
| Diabetes | < 0.01 | 0.77 |
| Antenatal_Antibiotics | 0.01 | 0.59 |

*Supplementary Table 3. Results (r2 and p value) of the envfit analysis from the vegan package.*

*
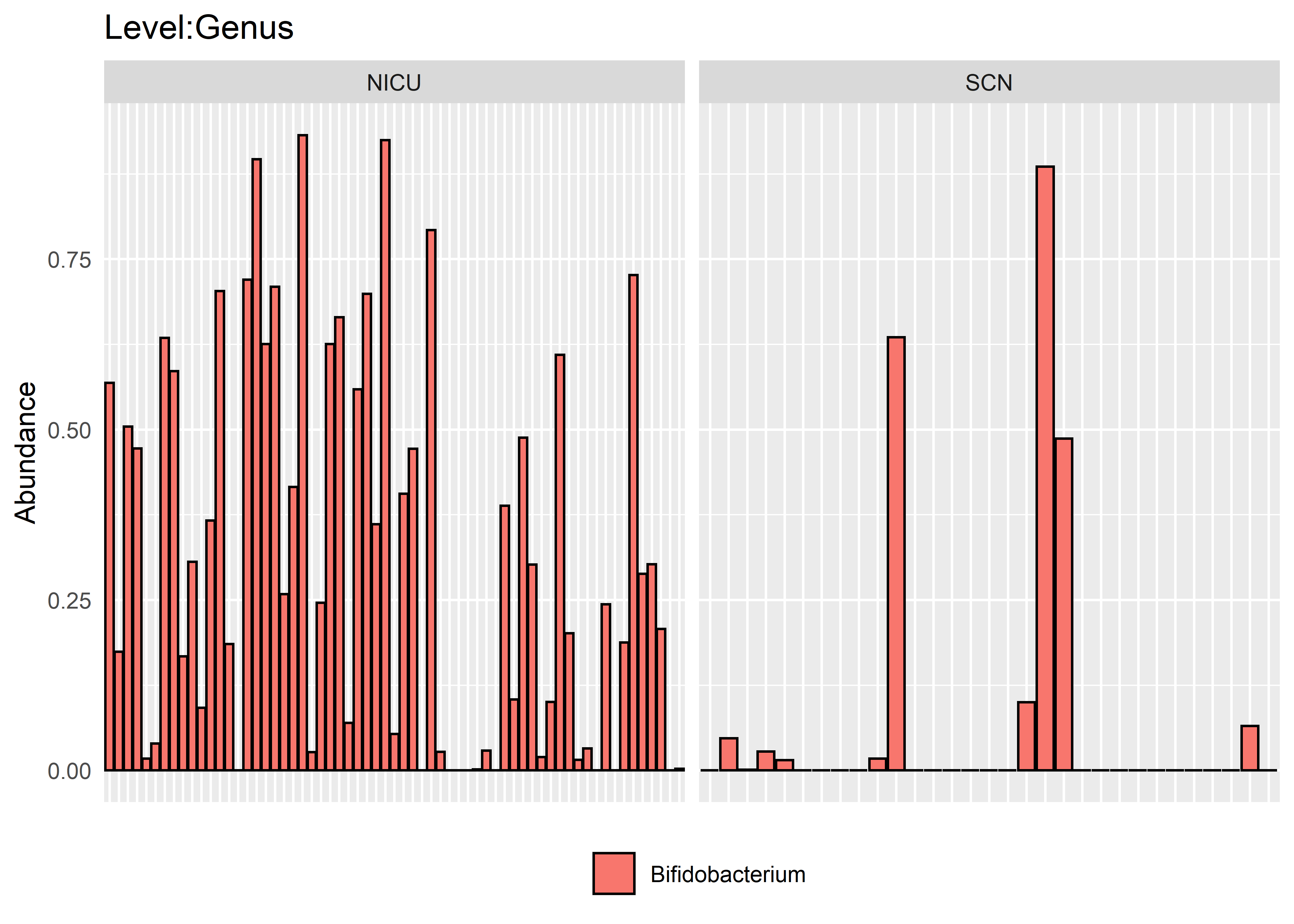
*

*Supplementary Figure 3. Bar chart representing the relative-abundance of Bifidobacterium across samples and between treatment groups, where NICU represents those treated with probiotics and SCN those not treated.*

*
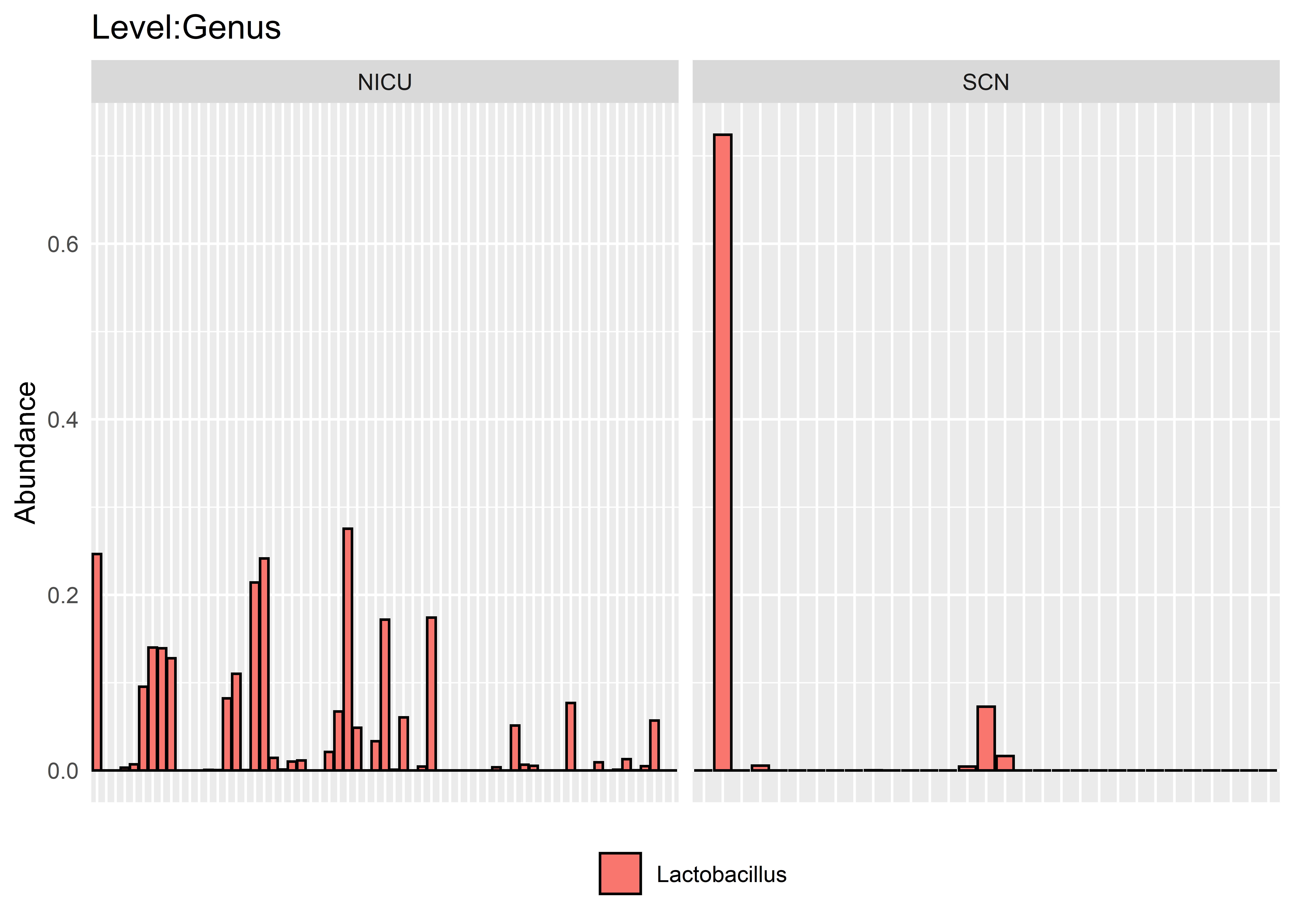
*

*Supplementary Figure 4. Bar chart representing the relative-abundance of Lactobacillus across samples and between treatment groups, where NICU represents those treated with probiotics and SCN those not treated.*

*
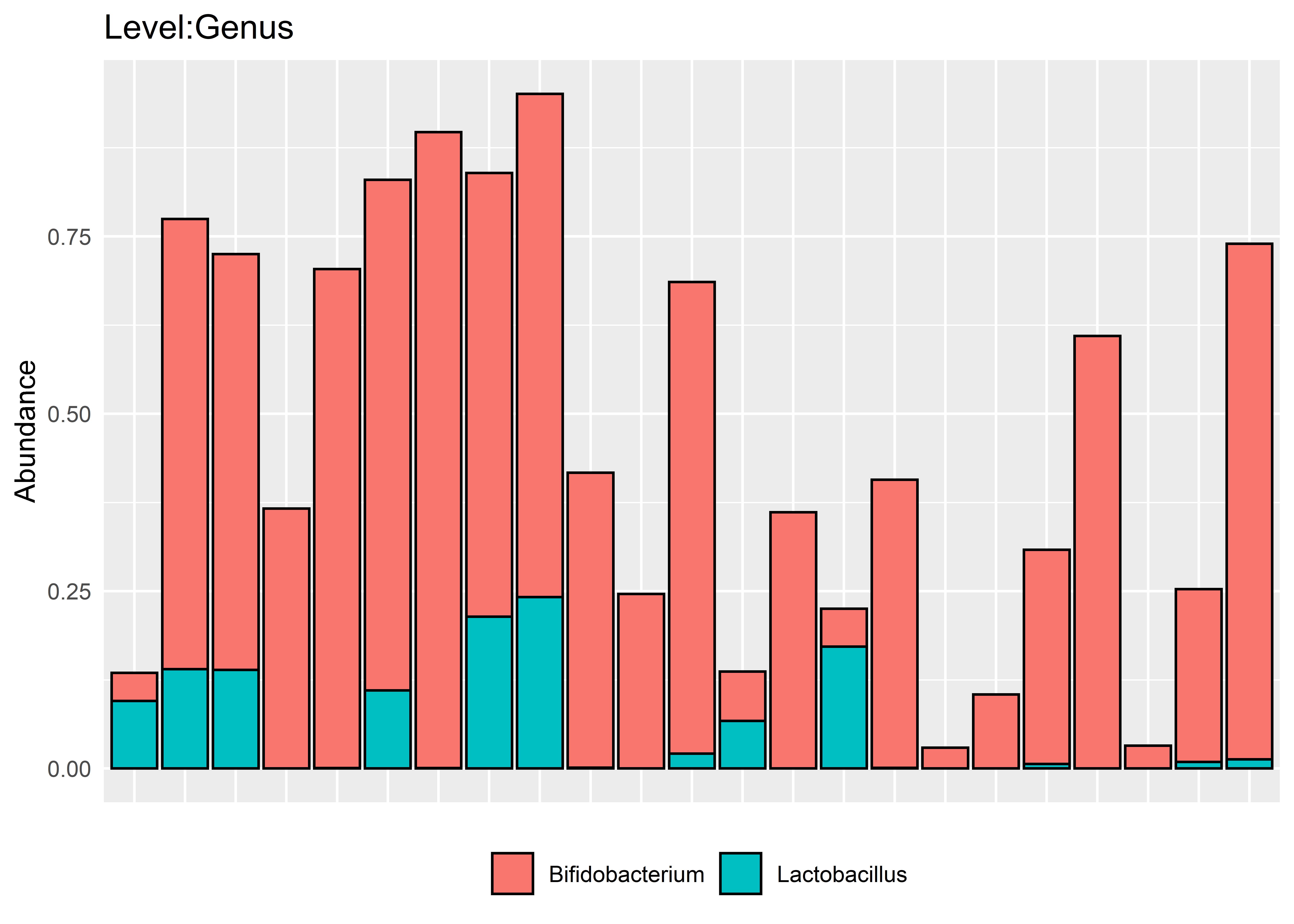
*

*Supplementary Figure 5. Bar chart representing the relative-abundance of Bifidobacterium and Lactobacillus across samples collected at > 36 weeks gestation (post probiotic-treatment) in the treatment group.*

*
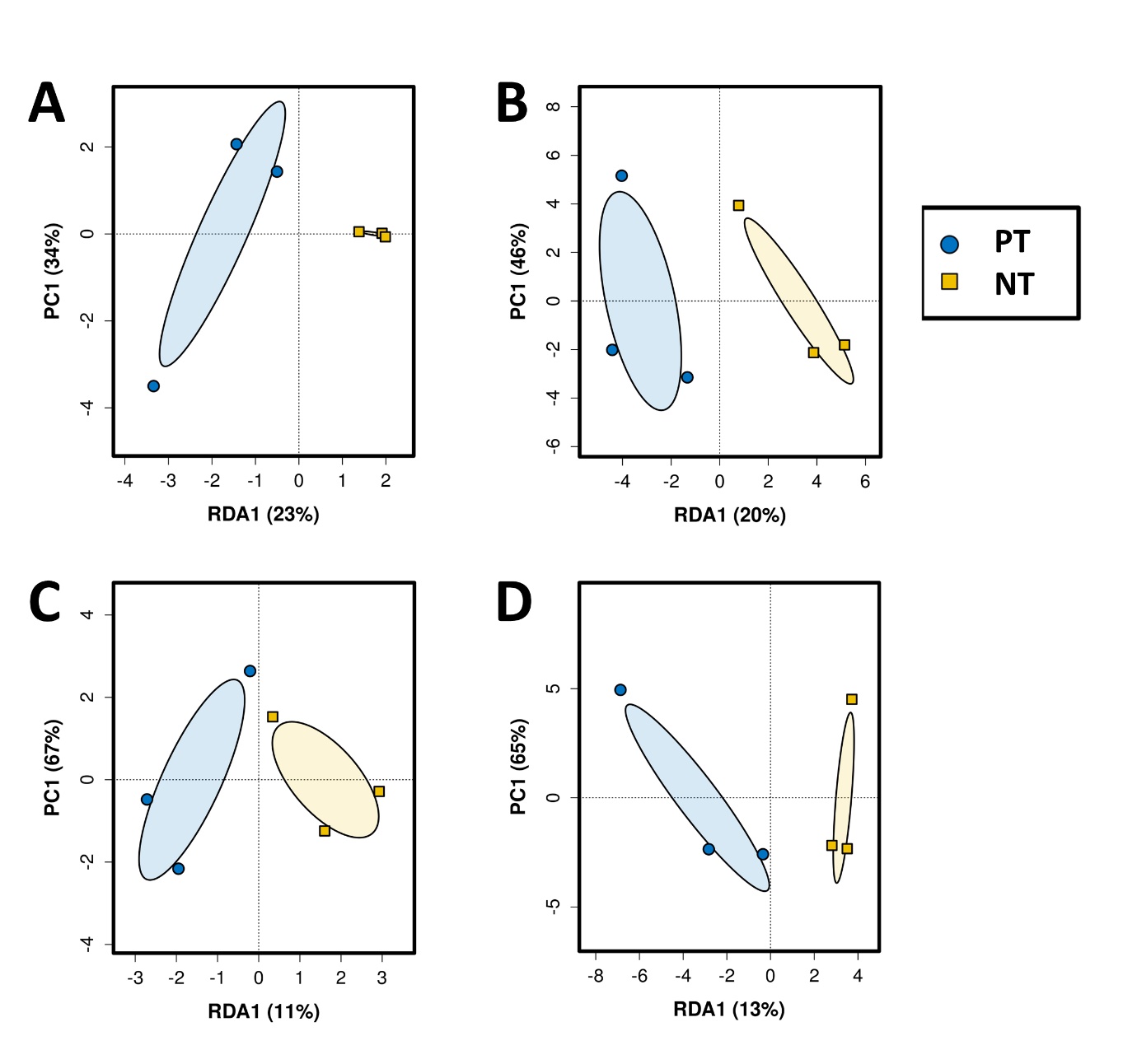
*

*Supplementary Figure 3. A: Redundancy analysis of clr transformed species-level taxonomic data, determined through shotgun metagenomics, and coloured by probiotic-supplemented (n = 3) and non-supplemented (n = 3) groups (P = 0.1), B: Redundancy analysis of clr transformed MetaCyc pathways data coloured probiotic-supplemented (n = 3) and non-supplemented (n = 3) (P = 0.6), C: Redundancy analysis of clr transformed MetaCyc groups identified using shotgun metagenomicsand coloured probiotic-supplemented (n = 3) and non-supplemented (n = 3) (P = 0.7), D: Redundancy analysis of clr transformed EC data identified through shotgun metagenomics coloured by probiotic-treated and non-treated groups (P = 0.5).* *Annotation for probiotic-treated; PT: non-treated; NT.*
